# Endoscopic submucosal dissection training: evaluation of an ex vivo training model with continuous perfusion (ETM-CP) for hands-on teaching and training in China

**DOI:** 10.1007/s00464-023-09940-9

**Published:** 2023-03-13

**Authors:** Jun Huang, Bing-ran Du, Wei-guang Qiao, Si-lin Huang, Lan-feng Xue, Liang Deng, Jun-ming Liang, Jun Wang, Jian-yi Li, Yu Chen

**Affiliations:** 1 Department of Gastroenterology, The Seventh Affiliated Hospital of Southern Medical University, No. 28, Liguan Road, Lishui Town, Nanhai District, Foshan, 528244 Guangdong China; 2 Department of Stomatology, Shunde Hospital, Southern Medical University (The First People’s Hospital of Shunde), Foshan, 528308 Guangdong China; 3 Department of Gastroenterology, Nanfang Hospital, Southern Medical University, Guangzhou, 510515 Guangdong China; 4 Department of Gastroenterology, South China Hospital, Medical School, Shenzhen University, Shenzhen, 518116 China; 5 Department of Gastroenterology, LunJiao Hospital, Shunde District, Foshan, 528244 Guangdong China; 6 Department of Gastroenterology, Xingtan Hospital Affiliated to Shunde Hospital of Southern Medical University, Foshan, 528325 China; 7 Department of Anatomy, School of Basic Medical Sciences, Southern Medical University, Guangzhou, 510515 Guangdong China; 8 The Third Affiliated Hospital, Southern Medical University, Guangzhou, 510515 Guangdong China; 9 Department of Anatomy, Guangdong Provincial Key Laboratory of Digital Medicine and Biomechanics, Guangdong Engineering Research Center for Translation of Medical 3D Printing Application, School of Basic Medical Sciences, Southern Medical University, Guangzhou, 510515 Guangdong China

**Keywords:** Endoscopic submucosal dissection, Training, Ex vivo model

## Abstract

**Background:**

The existing ex vivo models of endoscopic submucosal dissection (ESD) cannot simulate intraoperative hemorrhage well. We aimed to establish an ESD training method by applying an ex vivo training model with continuous perfusion (ETM-CP).

**Methods:**

Four training sessions were conducted for 25 novices under the guidance of 2 experts. Eventually, 10 novices completed ESD operations on a total of 89 patients after the training. The resection effectiveness, resection speed, complication rate, and novice performance before and after the training were compared. The data regarding the effects of the training and the model were gathered through a questionnaire survey.

**Results:**

In terms of the simulation effect of the model, ETM-CP was evaluated as similar to the live pig in all aspects (*P* > 0.05). The questionnaire analysis revealed that the ESD theoretical knowledge, skill operation, and self-confidence of novices were improved after the training (*P* < 0.05). The resection time per unit area had a correlation with the number of training periods (rs = – 0.232). For novice performance, the resection time per unit area was shortened (*P* < 0.05). There was no difference in patient performance between the novice group and the expert group after the training in terms of en bloc resection, R0 resection, complication rate, endoscopic resection bleeding (ERB) score, muscularis propria injury (MPI) score, and resection time per unit area (*P* > 0.05).

**Conclusion:**

The ETM-CP is effective for ESD training.

**Supplementary Information:**

The online version contains supplementary material available at 10.1007/s00464-023-09940-9.

Endoscopic submucosal dissection (ESD) was first carried out by Japanese expert Gotoda in 1999 [1]. Since then, ESD has evolved into a safe and effective endoscopic technique, which offers distinct advantages, such as enabling en bloc resection of large lesions, providing accurate pathological assessments, and reducing the risk of recurrence [2–4]. At present, ESD has been a standard treatment modality for early gastrointestinal lesions. As a safe, minimally invasive, and cost-effective treatment, ESD can reduce the pain and trauma of patients and improve the diagnosis rate of diseases [5, 6]. However, ESD is considered a technically difficult and time-consuming procedure, which requires a high operating ability of endoscopists [7, 8]. Hence, the wide application of ESD is hindered, especially in rural hospitals. The current ESD training mode in the world commonly adopts a series of training processes, such as clinical observation, tutor teaching, living animal training, close observation and learning, and independent clinical operation [9–11]. However, such a training mode presents a long training period, complex training process, and high training costs, so it is difficult to promote ESD training in rural hospitals in China. As a result, ESD technology in China is only available in large urban hospitals, and endoscopists in rural hospitals lack sufficient opportunities for clinical observation and practice. In addition, endoscopists in rural hospitals have relatively weak theoretical knowledge and also do not have enough time for systematic learning. Therefore, carrying out more systematic, professional, safe, economical, energy-saving, and effective ESD training in rural hospitals has become an urgent problem to be solved in China under the status of medical resource shortage.

To solve this issue, our team designed the ETM-CP. Four ESD training sessions were held within one year to facilitate rural endoscopists to participate in the training on weekends and other fragmented time. In each training session, well-known experts were invited to give lectures on ESD theoretical knowledge and operating experience. Meanwhile, we applied our self-developed, economical, easy-to-prepare, and effective isolated pig stomach model with continuous perfusion for practical ESD training. This training mode enables rural endoscopists to master ESD theoretical knowledge better. Also, endoscopists can have a deeper understanding of endoscopic operations and become more proficient in ESD through repeated training and practice on the ETM-CP. This training mode is conducive for rural endoscopists to gradually master ESD operations while reducing medical risks and economic costs.

## Materials and methods

### Generation of the ETM-CP

**Fig. 1:**
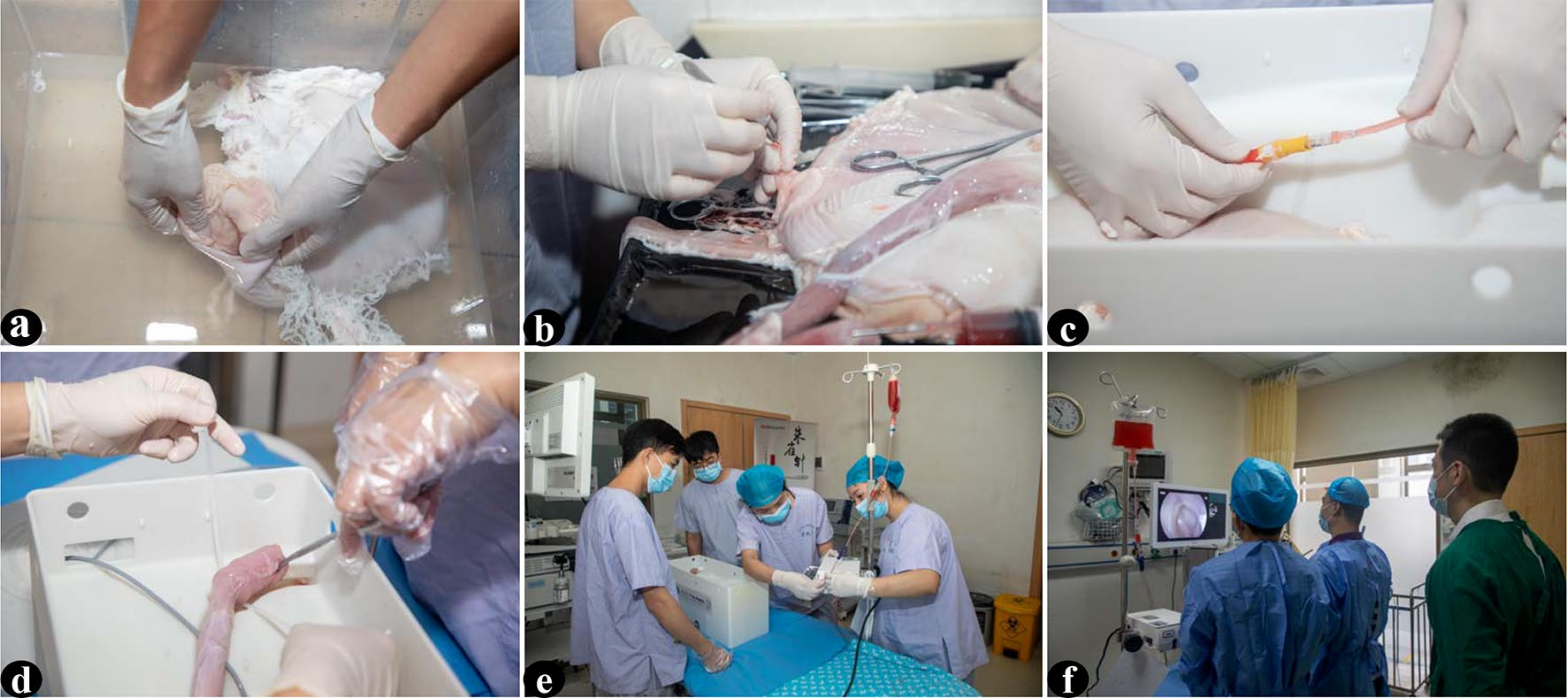
The process of making an ETM-CP. **a** The pig stomach was rinsed with 0.9% saline to clean off the mucus, dirt, and blood on the mucosal surface of the pig stomach and was then further cleaned with saline containing dimethicone oil and streptavidin to remove the mucus and air bubbles on the mucosal surface. **b** A part of the arteries and veins of the pig stomach was ligated and one aorta was separated. **c** The pig stomach was placed in the 3D-printed abdominal cavity simulation box in the left lateral position, and the separated aorta was connected to a perfusion bag containing 500 mL of artificially simulated non-coagulated blood. **d** The duodenal section was sutured, and the esophageal section was connected to the entrance of the 3D-printed abdominal cavity simulation box and the bag was fixed with nylon threads. **e** The perfusion bag was suspended to a certain height using an infusion rod and the infusion pump was used to adjust the perfusion pressure and drip rate. **f** The model was applied for ESD operations

**Fig. 2:**
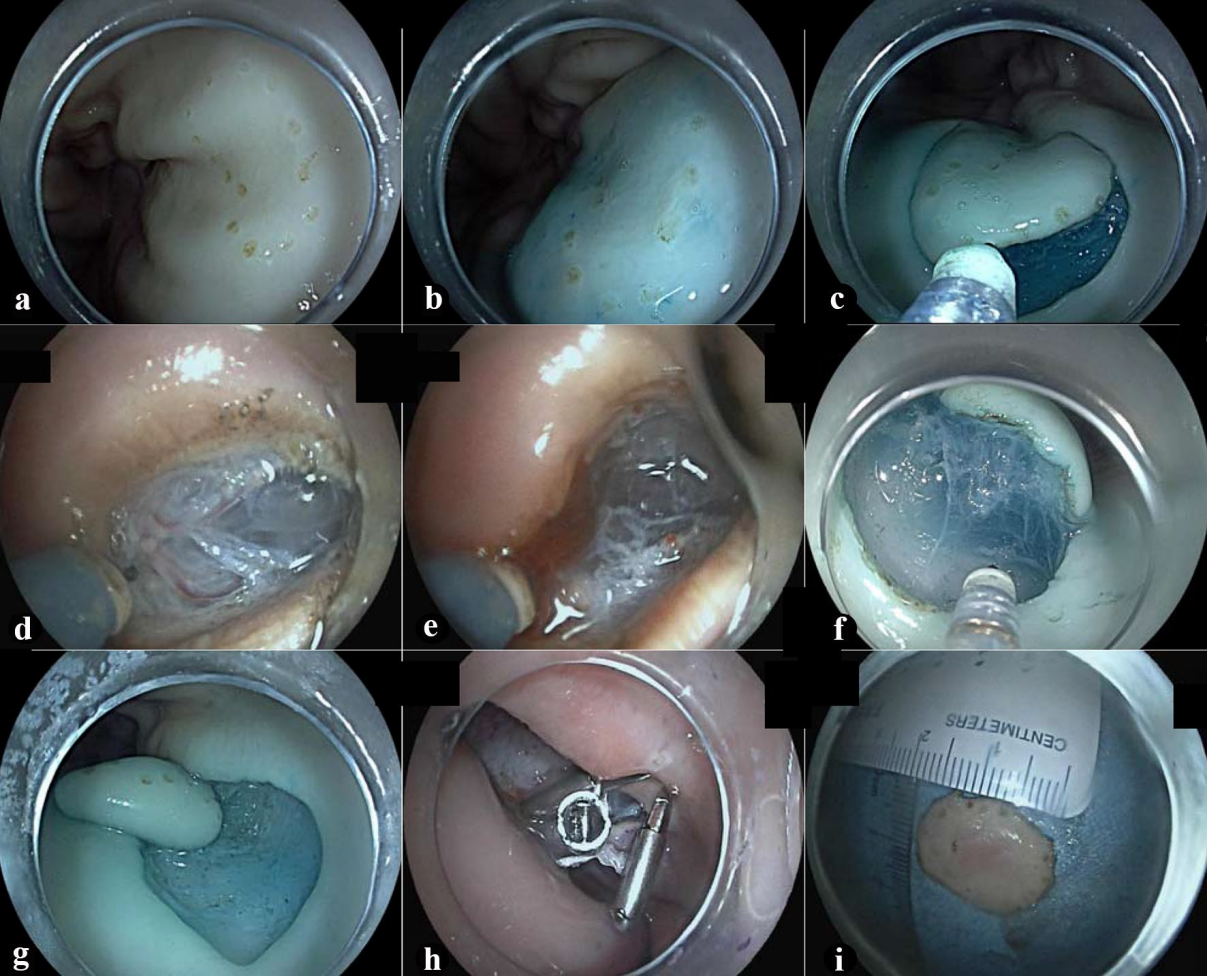
ESD procedures on the model. **a** Electrocoagulation was 3–5 mm away from the lesion margin to mark the extent of resection. **b** Multiple submucosal injections with Melan saline lateral to the lesion margin marking point were conducted to allow full elevation of the lesion and separation from the lamina propria. **c** Circumferential dissection was approximately 5 mm lateral to the lesion margin marking point. **d** Visualization of vessels. **e** Visible bleeding was observed after dissection of vessels. **f** The transparent cap technique with multiple repeated submucosal injections were used for complete submucosal debridement layer by layer. **g** Complete debridement of the submucosal lesion. **h** Suturing using a titanium clip. **i** Complete debridement of the isolated porcine mucosal epithelial specimen. In order to show the effect clearly, we selected the fine pictures taken in multiple operations to show

On the training day, fresh pig stomachs with esophagus of about 20–30 cm in length and duodenum of about 3–5 cm in length were purchased from the slaughter market. The pig stomach was rinsed with 0.9% saline to remove mucus, dirt, and blood on the mucosal surface. The mucus and air bubbles on the mucosal surface were further washed with saline containing dimethicone oil and streptavidin. The arteries and veins of the pig stomach were partially ligated. An aorta was separated and connected to a perfusion bag containing 500 mL of non-coagulated blood artificially simulated by a customized red pigmented liquid. A patent has been filed for this blood simulation technology. The perfusion bag was suspended to a certain height using an infusion pole. The perfusion pressure and drip rate were controlled using an infusion pump to maintain continuous perfusion of the isolated pig stomach. The pig stomach was fixed with a specially designed 3D-printed abdominal cavity simulation box. The pig stomach was placed in a way that simulated the left lateral recumbent position of the human body. The duodenal dissection was ligated with sutures. The esophageal dissection was attached to the entrance of the 3D-printed abdominal simulation box and fixed with nylon thread. The pig stomach was placed in the left lateral recumbent position for simulating gastroscopic assistant operation. The electrode sheet was applied to the surface of the pig stomach. The 3D-printed simulated abdominal cavity box lid was covered to preserve certain closures (as shown in Figs. 1 and 2).

### Assessment of the ETM-CP by the rural endoscopists

Before the training, a questionnaire for ESD training was issued to rural endoscopists in Foshan City, Guangdong Province. Meanwhile, the existing ESD training models were investigated. ESD training was performed by virtue of the ETM-CP. Participants were required to complete a questionnaire to evaluate the ETM-CP using the Likert scale, involving blood simulation, texture and color, anatomical level, freedom of movement of instruments, tactile feedback during operation, the realism of the simulation training, and overall satisfaction. The participants’ evaluation of the ETM-CP was compared with their evaluation of silicone simulation models and live pigs.

### Participants

In this study, a total of 29 novices and 2 experts participated in ESD training sessions from September 25, 2020 to December 5, 2021. Eventually, 25 novices completed the training. 15 novices did not perform clinical ESD because of the lack of appropriate clinical cases. And 10 novices successfully performed clinical ESD independently after the training. These 10 novices were eventually included in the novice group. The 15 novices who did not perform clinical ESD were removed from the novice group. The novice group had an average of 9.40 years of experience in endoscopy. The number of gastroscopy procedures performed by the novice group was more than 1000. 80% of the novice group performed more than 2000 gastroscopy procedures. Only 20% of the novice group had 0 to 5 ESD experiences. 80% of the novice groups did not have experience in ESD operation before training (as shown in Table S1). 2 experts were included in the expert group. The 2 experts were endoscopists who had independently performed more than 500 cases of ESD procedures.

### Training plan and assessment

The participants received four sessions of ESD training each year in fragmented time, such as weekends. The training contents of each session included both theoretical lectures and practical ESD operation teaching. The expert group performed a complete demonstration of standardized ESD operation on the model (as shown in Fig. 2). The novice group completed observation and learning sessions. After theoretical study and practical observation, the novice group received operation training and tried to carry out ESD on the ETM-CP independently. A pig stomach can be reused many times. The novice can repeat ESD operations on the ETM-CP. A novice performed ESD, while another novice acted as an assistant. Each novice can actually perform ESD independently about 5 to 8 times in 1 day. Each pig stomach can be used to perform approximately 10 ESD operations.

Before the training, a questionnaire survey on the ESD theoretical knowledge mastery, operational proficiency, and self-confidence was conducted for the novice group, followed by theoretical knowledge teaching, operation skill explanation, expert surgery video broadcasting, and case sharing. The specific contents of the training sessions are shown in Table S2.

At the end of the four training sessions, a questionnaire on ESD theoretical knowledge, operational proficiency, and self-confidence was administered to the novice group again. The questionnaire was scored on the Likert scale. The differences in the questionnaire concerning ESD theoretical knowledge, operational proficiency, and self-confidence before and after the training were analyzed.

After completing each training session, the trainees in the novice group returned to their local hospitals and performed ESD operations. And 10 novices successfully performed clinical ESD. The novice group required a nurse as an assistant and an anesthesiologist to be present when performing clinical ESD. 2 novices completed clinical ESD in the presence of an expert and the remaining 8 novices all completed clinical ESD without the presence of an expert. The information of patients receiving ESD performed by doctors in the novice group and the expert group from 2020 to February 1, 2022 were collected. The number of cases in the novice group that eventually completed clinical ESD after training was 8, 12, 6, 8, 14, 8, 7, 3, 5, and 11, respectively. A comparison was made between the novice group and the expert group on the completion of ESD operations, including en bloc resection, R0 resection, complications, and adverse events. The number of training periods required by unit area resection was compared in the novice group. Moreover, the differences between the novice group and the expert group in the ESD resection time per unit area, endoscopic resection bleeding (ERB) score, and muscularis propria injury (MPI) score were compared. Further, the clinical application of ESD after the training was evaluated.

### Statistical analysis

SPSS 26.0 was applied for statistical analysis. The measurement data were expressed as (mean ± standard deviation) or (median, interquartile spacing), and the *t test* was used for comparisons between the two groups. The *non-parametric test* was used for non-normally distributed data. The count data were expressed as frequencies, and the *chi-square test* was used for comparisons between the two groups. *Spearman rank correlation coefficient* was used to assess the correlation between the two groups of data. The test level was *α* = 0.05.

## Results

### Evaluation of the ETM-CP

**Fig. 3:**
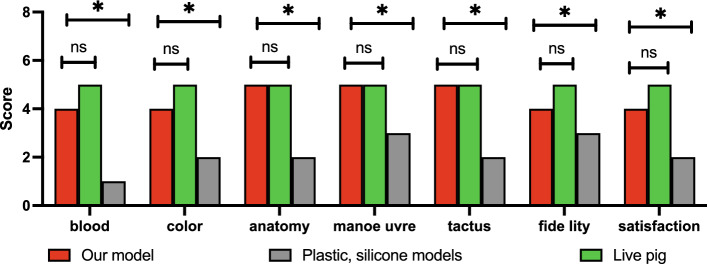
Comparisons between the ETM-CP and plastic silicone models and live pigs in terms of simulation of blood, texture and color, anatomical level, freedom of movement of instruments, tactile feedback during operation, the realism of simulation training, and overall satisfaction. *P < 0.05

The simulation degree of the ETM-CP was rated as worst, worse, average, better, and best, with a score range of 1–5. According to the results of 19 valid questionnaires, the mean score of the ETM-CP was 4, 4, 5, 5, 5, 5, 4, 4, and 4 in terms of simulation of blood, texture and color, anatomical level, freedom of movement of instruments, tactile feedback during manipulation, the realism of simulation training, and overall satisfaction. The live pig model scored 5 on the simulation of blood, texture and color, anatomical level, freedom of instrument movement, tactile feedback during manipulation, the realism of simulation training, and overall satisfaction. The silicone simulation model scored 1, 2, 2, 3, 2, 3, 3, 2, and 2 in terms of blood simulation, texture and color, anatomical level, freedom of movement of instruments, tactile feedback during operation, the realism of simulation training, and overall satisfaction. There was no statistical difference in various aspects between the ETM-CP and the live pig model. The ETM-CP was superior to the silicone simulation model in all aspects with statistical significance (as shown in Fig. 3).

### Questionnaire review

**Fig. 4:**
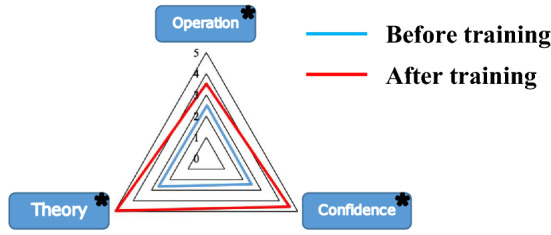
Comparisons of questionnaire scores on three dimensions of ESD theoretical knowledge, operational proficiency, and self-confidence of the novice group before and after the training. *P < 0.05

The questionnaire on ESD theoretical knowledge, operational proficiency, self-confidence, and gastroscope proficiency was distributed to the novice group. Finally, 14 valid questionnaires were received. The questionnaire was evaluated by the Likert scale, and the results showed that the subjective proficiency of the respondents in gastroscope operations was increased after the training compared with that before the training (4.86 ± 0.378 vs 4.29 ± 0.756, *P* < 0.05). Compared with the scores before the training, the scores of novices’ subjective assessment in the three dimensions of ESD theoretical knowledge mastery, skill operation mastery, and self-confidence were improved after the training, with scores of (2.64 ± 0.93 vs 4.93 ± 0.27), (2.5 ± 0.94 vs 4.93 ± 0.27), and (2.43 ± 1.01 vs 4.55 ± 0.46) (as shown in Table S3). These differences were statistically significant (*P* < 0.001), respectively (as shown in Fig. 4).

### Training assessment

The data of 122 patients undergoing ESD performed by the novice group (*n* = 10) and expert group (*n* = 2) from 2020 to February 1, 2022 were collected. The novice group carried out 89 cases of ESD operations and the expert group carried out 33 cases of ESD operations. The gender composition of the patients operated by the two groups was 45:44 and 23:10, and the difference was not statistically significant. The mean age of the patients operated by the two groups was 54.0 ± 13.3 and 56.6 ± 11.3, and the difference was not statistically significant. The lesion distribution and depth distribution of the patients operated by the two groups were not statistically significant. The median lesion size of the patients operated by the two groups was 225.0 mm^2^ and 240 mm^2^, and the difference was not statistically significant (as shown in Table 1). The endoscopic en bloc resection rate was 100% in both groups. The difference in the R0 resection rate between the two groups was not statistically significant (98.9% vs 100.0%, *P* > 0.05). The difference in the incidence of complications and perforation rate was not statistically significant (6.7% vs 6.1%, 6.7% vs 6.1%, *P* > 0.05). No postoperative bleeding occurred in the two groups (as shown in Table 1).

**Table 1:** Comparisons of the ESD patients in the novice group and the expert group

Variables	Novice group (*n* = 89)	Expert group (*n* = 33)	*P*
Gender, *n* (%)			0.059
Male	45 (50.6)	23 (67.6)	
Female	44 (49.4)	10 (32.4)	
Age, mean ± SD (y)	54.0 ± 13.3	56.6 ± 11.3	0.297
Tumor location, *n* (%)			0.137
Stomach	46 (51.7)	15 (45.5)	
Right colon	7 (7.9)	7 (21.2)	
Left colon	36 (40.4)	11 (33.3)	
Depth, *n* (%)			0.108
M	37 (41.6)	20 (60.6)	
SM	41 (46.1)	12 (36.4)	
Muscularis propria	11 (12.4)	1 (3.0)	
Tumor size, median and interquartile ranges (mm^2^)	225.0 (100 ~ 430)	240.0 (100 ~ 550)	0.961
Endoscopic complete resection, *n* (%)	89 (100.0)	33 (100.0)	-
Histological complete resection, *n* (%)	88 (98.9)	33 (100.0)	1.000
Complication, *n* (%)	6 (6.7)	2 (6.1)	1.000
Postprocedure bleeding, *n* (%)	0 (0.00)	0 (0.00)	-
Perforation, *n* (%)	6 (6.7)	2 (6.1)	1.000
Procedure time, median and interquartile ranges (min/mm^2^)	0.29 (0.14 ~ 0.63)	0.18 (0.10 ~ 0.52)	0.109
ERB score, median and interquartile ranges	2.0 (1 ~ 3)	1.0 (1 ~ 2)	0.094
MPI score, median and interquartile ranges	1.0 (1 ~ 1)	1.0 (1 ~ 2)	0.518

ERB score: endoscopic resection bleeding score; ERB-0 = 1, ERB-c (controlled) = 2, ERB-c1 = 3, ERB-c2 = 4, ERB-c3 = 5, and ERB-unc (uncontrolled) = 6; MPI score: muscularis propria injury score; MPI-0 = 1, MPI-ia = 2, MPI-ib = 3, MPI-pa = 4, and MPI-pb = 5

**Fig. 5:**
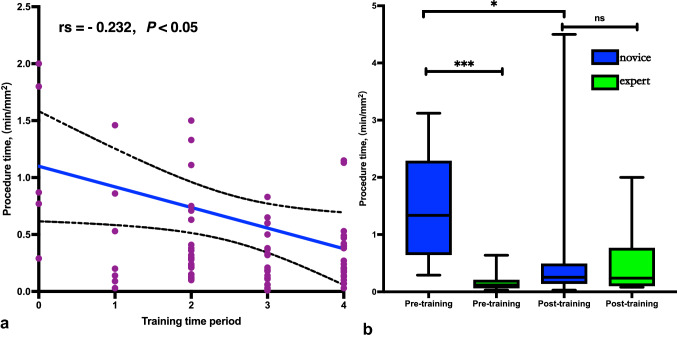
Evaluation of the training effectiveness of trainees. **a** Variation process of excision time per unit area in the novice group as the training phase progresses. **b** Changes in excision time per unit area in the novice group before and after the training, and comparison with the expert group. Procedure time: operation time/tumor area (min/mm^2^). ***P < 0.0001, *P < 0.05, rs: Spearman rank correlation coefficient = -0.232

As the progressive progression from the 1st to the 4th training session, the novice group consisting of 10 rural endoscopists showed a gradual decrease in the resection time per unit area, and the resection time per unit area had a negative correlation with the number of training sessions (*Spearman rank correlation coefficient* = − 0.232, *P* < 0.05). Before the training, the median resection time per unit area in the novice group was longer than that in the expert group (1.34 vs 0.12, *P* < 0.001). After the training, the median resection time per unit area in the novice group was significantly shorter than that before the training (1.34 vs 0.26, *P* < 0.05) and initially reached the level of resection time per unit area in the expert group (0.26 vs 0.24, *P* > 0.05) (as shown in Fig. 5).

The median ESD resection time per unit area for the 89 patients operated by the novice group and the 33 patients operated by the expert group was 0.29 min/mm^2^ vs 0.18 min/mm^2^ (*P* = 0.109), and the difference was not statistically significant. The median ERB score was 2.0 vs 1.0 (*P* = 0.094), and the difference was not statistically significant. The median MPI score was 1.0 vs 1.0 (*P* = 0.518), and the difference was not statistically significant (as shown in Table 1).

## Discussion

ESD as a safe and minimally invasive procedure to remove gastrointestinal lesions [12] is becoming a viable treatment modality for the resection of early gastrointestinal cancer. However, due to its relatively steep learning curve, complex operations, and high operative risk, ESD has not been widely popularized and practiced [13]. Our team constructed a training system for rural endoscopists on ESD by applying our self-developed ETM-CP. The results showed that rural endoscopists with non-senior titles had fewer opportunities to complete ESD operations. Rural endoscopists, especially those with non-senior titles, did not receive much ESD training. In addition, the questionnaire results showed that most of the ESD training models available to rural endoscopists in China were live pigs and silicone simulation models. A majority of rural endoscopists lacked training experience with various ESD models. Rural hospitals also did not have appropriate space, anesthesia and feeding conditions, and live pig sources to conduct effective ESD training. The Likert scale questionnaire showed that the application of our self-developed model to ESD theoretical teaching and ESD practical training in fragmented time can effectively improve the trainees’ proficiency in gastroscope operation, ESD theoretical knowledge, operation ability, and self-confidence. The novice group was competent to perform ESD after a series of training. The novice group and the expert group carried out a total of 122 cases of ESD operations and both two groups achieved en bloc resection. The R0 resection rate of the novice group was 98.9%, and the incidence of adverse events was less than 10%, with no statistical difference compared to the expert group with rich experience in ESD operation. During the training period, the ESD resection time per unit area in the novice group decreased as the number of training periods increased, indicating that the ESD operation proficiency of the novice group was gradually improving as the training progressed. Although the correlation coefficient was only 0.232, it can still indicate that ETM-CP was somewhat useful for ESD training. It demonstrated a progressive trend in the novice group. In addition, we noticed some differences in the patient characteristics of each training session (as shown in Table S4). There was a statistically significant difference in the sex and depth of lesions between each training session. Also, there was no statistical difference in the comparison of 4 training sessions in terms of age, tumor location, and tumor size. We believed that the factors that have a greater impact on the difficulty of ESD operation were location and size. However, this study was conducted as 1 single-center, small sample study. The small sample size may lead to bias brought about by differences in tumor depth. This may have led to the result of a small correlation coefficient. At the end of the final training, there was no statistical difference between the novice group and the expert group in terms of ESD resection time per unit area and complication control. This was a good indication that ETM-CP was effective for ESD training.

The previous study has demonstrated that ESD can be safely performed in small clinics with established therapeutic methods in Japan [14]. The ESD training system currently used in Japan has gone through the following stages: theoretical learning and case observation, training and learning as an expert assistant, development of ESD for early gastric lesions, development of ESD for the rectum, and development of ESD for the colon and esophagus [15, 16]. This traditional classical training system often takes at least 3 to 4 years to initially train a qualified ESD operator, which is inconsistent with the current urgent need to promote the application of ESD in rural hospitals economically and effectively [17, 18]. Some novel ESD electronic simulation learning programs can be used for repeated learning and practice of ESD [19, 20], but pure mechanical simulation and virtual reality models are limited in advanced endoscopic resection training because of their high cost and inability to reproduce tissue elasticity and provide tactile feedback [21–23]. The consensus among ESD experts is that the main ESD operators and their assistants must receive four stages of standardized technical training: gastric ESD learning, field observation, animal experiments, and formal operation [24, 25]. Among them, animal experimental models are divided into two types: ex vivo animal models and in vivo animal models. The domestic pig is currently recommended as an optimal animal model for ESD training because its anatomical structure is similar to that of the human digestive tract [26, 27]. However, due to the limitations of the source, cost, feeding, anesthesia, preparation conditions, and non-reusability of live pigs, only a small number of tertiary hospitals in major cities in China can use live pigs as an animal model for ESD training. The ex vivo animal model commonly used is the ex vivo pig stomach, with the limitations of lacking blood perfusion, stimulation of heart pulsation, respiration, and peristalsis of the digestive tract, lacking obvious lifting signs, causing excessive mucus secretion, lacking physiological response to injection and electrocoagulation, etc. [27–29]. Some experts recommend that in vivo animal models should be used for training on the basis of proficient operation of ex vivo animal models, which is more conducive to the improvement of clinical practice.

Our team innovatively developed a new ex vivo training model of isolated pig stomachs with continuous perfusion. We isolated one of the blood supply arteries through ligation of the main blood supply artery of the pig stomach [30]. The pressure perfusion pattern was used for continuous perfusion to maintain the blood flow status of the pig stomach. The perfusion pressure and flow rate were controlled by the suspension at a certain height with an infusion pump. Finally, the results showed that the submucosal vessels were visible during ESD operation (bleeding effect as shown in the Supplementary Video). This model can simulate the clinical bleeding effect, which is suitable for practicing ESD hemostasis, electrocoagulation, and other operations. In addition, a closed 3D printing box was used to simulate the anatomical direction of the human body in the left lateral recumbent position, and the position change could be adjusted appropriately, which is closer to the normal clinical state. The ETM-CP can simulate intraoperative bleeding in ESD. Novices can gradually master hemostasis skills through repeated hemostasis training on the ETM-CP. Adequate experience in hemostasis allows the novice to stop bleeding more quickly when encountering intraoperative bleeding in ESD. Preparation of the ETM-CP requires obtaining fresh isolated porcine stomach and preparation of the model. We usually go to the slaughterhouse at 6:00 a.m. to obtain fresh isolated pig stomachs. Then, we soak the pig stomach in 9% saline for preservation and transportation. After that, the model is prepared according to the steps described previously. The preparation of the model takes about 1 h. We arrange 2 to 3 people to go for the preparation of ETM-CP. We can prepare 5 ETM-CP models in 3 h. The ETM-CP is reusable. Preparation of ETM-CP takes very little time and effort compared to live pig. At the same time, the ETM-CP has a bleeding simulation that other ex vivo models do not have.

At present, the traditional endoscopy training mode is mostly observation learning. After acquiring sufficient theoretical knowledge, experienced experts lead the teaching, and the novices often learn as assistants. After a long time and a large number of clinical cases, the novice starts to perform endoscopic surgery as the chief surgeon under the guidance of experts. Eventually, the novice is able to perform the procedure independently in the clinic. This endoscopy training mode usually requires a long training cycle and a large number of clinical cases [18]. Hence, such training mode is only applicable to affiliated hospitals and teaching hospitals with abundant teaching resources and cases, but rural hospitals do not have such medical and teaching resources, which seriously hinders the training of qualified endoscopic surgeons and also the development of endoscopic technology in Chinese rural hospitals. Encouragingly, many new training models developed in recent years can achieve multiple endoscopic operations in a relatively short time [31–33], which contribute to expediting the learning curve, increasing the operational experience, and shortening the period of training to some extent. Some studies have also shown that repeated in vitro model practice can shorten the operating time under endoscopy and improve operating proficiency. The traditional training mode adopts a phased continuum of training with a gradual exercise and cumulative learning curve, and the training cycle of such mode usually takes 3 to 5 years. Since endoscopic operations have certain muscle memory, some studies try to conduct short-term 1-day training by applying novel ex vivo animal models [34, 35]. Repeated operations in a short period of time can reduce the operation time of trainees with the increase of the number of operations, which indicates that appropriate use of short-term training can achieve certain training effects. What the endoscopists in China’s rural hospitals lack is not the way to learn the theoretical knowledge of endoscopy, but the opportunity for clinical observation and practice. The traditional training mode relying on a large number of clinical cases. Endoscopy training in rural hospitals in China lacks a highly simulated training model that can save time and cost and facilitate repeated practice in a short period of time. Our team created the ETM-CP, which can mimic intraoperative bleeding in ESD, and can be used for training in ESD.

The ETM-CP confers a new, economical, and effective model for ESD training, which overcomes the limitations of traditional ESD training, such as a long training period, high cost of acquiring live pigs, and insufficient blood perfusion in ex vivo. Our study was clinically validated and showed the usefulness of ETM-CP for ESD training. However, a multicenter, large sample study is still needed to further precisely. This training system can enable rural endoscopists to gradually become familiar with and master ESD operations under the premise of controlling medical risks and economic costs. However, this training system also has limitations. For example, the sources of freshly isolated pig stomachs are not uniform. The model lacks the simulation of cardiac pulsation, respiration, and peristalsis of the digestive tract, the physiological response to injection and electrocoagulation, and the simulation of colorectal lesions. Moreover, the training system lacks the observation and learning of clinical operations by Japanese experts.

## Supplementary Information

Below is the link to the electronic supplementary material.

Supplementary file1 (DOCX 25 KB)

Supplementary file2 (MP4 3292 kb)
