# Supplementary material for: Endoscopic submucosal dissection training: evaluation of an ex vivo training model with continuous perfusion (ETM-CP) for hands-on teaching and training in China
Source: Surg Endosc. 2023 Mar 13;37(6):4774–83. doi: 10.1007/s00464-023-09940-9 (PMC10234865; doi:10.1007/s00464-023-09940-9)
Supplement: Supplementary file 1 — Supplementary file1 (DOCX 25 KB) [file 464_2023_9940_MOESM1_ESM.docx]

**Supplementary**

**Table. S1** Pre-training information for novice group

| Variables | Novice group(n=10) |
| --- | --- |
| Time to carry out endoscopy, mean ± SD (years) | 9.40 ± 4.50 |
|  |  |
|  |  |
| Number of ESD, n (%) |  |
| 0 | 8 (80) |
| 0～5 | 2(20) |
| 5~10 | 0 (0) |
| 10~50 | 0 (0) |
| ≧50 | 0 (0) |
| Number of gastroscopy, n (%) |  |
| 100～500 | 0 (0) |
| 500~1000 | 0 (0) |
| 1000~2000 | 2 (20) |
| >2000 | 8 (80) |

**Table. S2** Course titles of the training

| Training | Course title |
| --- | --- |
| The first training | Recognition of early gastric cancer after sterilization |
|  | Endoscopic suture technique |
|  | NICE classification and JNET classification of intestinal lesions |
|  | White light screening for early gastric cancer and basic techniques of ESD |
| The second training | Explanation of the Kyoto classification of gastritis |
|  | Practical treatment skills under digestive endoscopy in rural hospitals |
|  | Nursing cooperation and introduction of consumables for treatment under digestive endoscopy |
|  | Rebroadcast of surgery demonstration |
| The third training | Endoscopic screening for early gastric cancer and management of indications for endoscopic therapy |
|  | Technical strategies for colorectal ESD |
|  | Electric knife mode selection |
|  | Experience in the endoscopic and pathological diagnosis of autoimmune atrophic gastritis |
| The fourth training | “Super G” Detective Salon |
|  | Interpretation of guidelines for endoscopic treatment of intestinal lesions |
|  | Prevention and treatment of intestinal ESD complications |
|  | Research progress of the clinical application of LCI in the upper gastrointestinal tract |
|  | Endoscopic traction and suture techniques |

**Table. S3** Comparisons of gastroscopy proficiency, ESD theoretical knowledge, operational proficiency, and self-confidence of the novice group before and after the training

| Variables | Pre-training (n=14) | Post-training (n=14) | *P* |
| --- | --- | --- | --- |
| Gastroscopy subjective proficiency score, mean ± SD | 4.29 ± 0.76 | 4.86 ± 0.38 | 0.03 |
| Theory score, mean ± SD | 2.64 ± 0.93 | 4.93 ± 0.27 | < 0.001 |
| Operation score, mean ± SD | 2.50 ± 0.94 | 3.52 ± 0.68 | < 0.001 |
| Confidence score, mean ± SD | 2.43 ± 1.01 | 4.55 ± 0.46 | < 0.001 |

Gastroscopy subjective proficiency score: subjective judgment of gastroscopists on the ability of gastroscopy.

**Table. S4** Patient characteristics of the novice group for each training session

| Variables | 1st(n=9) | 2nd (n=27) | 3rd(n=23) | 4th (n=24) | *P* |
| --- | --- | --- | --- | --- | --- |
| Sex, n (%) |  |  |  |  | 0.026 |
| Male | 6(66.7) | 9 (33.3) | 9(39.1) | 17 (70.8) |  |
| Female | 3(33.3) | 18 (66.7) | 14(60.9) | 7 (29.2) |  |
| Age, median (y) | 56 | 53 | 52 | 50 | 0.509 |
| Tumor location, n (%) |  |  |  |  | 0.934 |
| Stomach | 2(22.2) | 10 (37.0) | 7(30.4) | 9 (37.5) |  |
| Right colon | 6(66.7) | 13 (48.1) | 14(60.9) | 13 (54.2) |  |
| Left colon | 1(11.1) | 4 (14.9) | 2(8.7) | 2 (8.3) |  |
| Depth, n (%) |  |  |  |  | 0.015 |
| M | 2(22.2) | 17 (63.0) | 12(52.2) | 6 (25.0) |  |
| SM | 4(44.5) | 9 (33.3) | 7(30.4) | 16 (66.7) |  |
| Muscularis propria | 3(33.3) | 1 (3.7) | 4(17.4) | 2 (8.3) |  |
| Tumor size, median (mm^2^) | 288 | 225 | 360 | 225 | 0.256 |
